# Characterization of *LGR5* expression in poorly differentiated colorectal carcinoma with mismatch repair protein deficiency

**DOI:** 10.1186/s12885-020-06791-8

**Published:** 2020-04-15

**Authors:** Tomoyuki Nakajima, Takeshi Uehara, Mai Iwaya, Yukihiro Kobayashi, Yasuhiro Maruyama, Hiroyoshi Ota

**Affiliations:** 1 Department of Laboratory Medicine, Shinshu University School of Medicine, 3-1-1 Asahi, Matsumoto, 390-8621 Japan; 2 Department of Gastroenterology, Suwa Red Cross Hospital, Suwa, Japan; 3 Department of Biomedical Laboratory Medicine, Shinshu University School of Medicine, Matsumoto, Japan

**Keywords:** Leucine-rich repeat-containing G-protein-coupled receptor 5, Mismatch repair, Poorly differentiated colorectal carcinoma, RNA in situ hybridization

## Abstract

**Background:**

Leucine-rich repeat-containing G-protein-coupled receptor 5 (LGR5) is a promising intestinal stem cell and carcinoma stem cell marker. We examined the relationship between mismatch repair (MMR) protein deficiency and *LGR5* expression in poorly differentiated (PD) colorectal carcinoma (CRC).

**Methods:**

In 29 cases of PD-CRC, deficiencies in MMR proteins (MLH1, PMS2, MSH2, MSH6) and β-catenin expression were identified by immunohistochemistry (IHC). *LGR5* expression was examined by the RNAscope assay in tissue microarrays.

**Results:**

*LGR5* H-scores in MMR-deficient (MMR-D) cases were significantly lower than those in MMR-proficient (MMR-P) cases (*P* = 0.0033). Nuclear β-catenin IHC scores in MMR-D cases were significantly lower than those in MMR-P cases (*P* = 0.0024). In all cases, there was a positive correlation between *LGR5* H-score and nuclear β-catenin IHC score (r = 0.6796, *P* < 0.001). Even in MMR-D and MMR-P cases, there was a positive correlation between *LGR5* H-score and nuclear β-catenin IHC score (r = 0.7180, *P* < 0.0085 and r = 0.6574, *P* < 0.003, respectively). MMR-D CRC cases showed low expression of *LGR5*, which may be due to low activation of the Wnt/β-catenin signaling pathway.

**Conclusions:**

Our results reveal the relationship between *LGR5* expression and MMR protein profiles in PD-CRC. A further study is warranted to confirm these findings.

## Background

Leucine-rich repeat-containing G-protein-coupled receptor 5 (LGR5) is a promising intestinal stem cell marker [1]. LGR5 is also a candidate marker for colorectal cancer (CRC) stem cells [2] and may be closely involved in the progression and prognosis of CRC [3]. However, Jang et al. suggested a suppressive role for *LGR5* in CRC progression [4].

CRC is one of the most common carcinomas worldwide [5] and is graded into well, moderately, and poorly differentiated types according to gland structures. The colon carcinogenesis model is roughly divided into an adenoma–carcinoma sequence and a serrated neoplasia pathway involving microsatellite instability (MSI) [6] [7]. Approximately 70–80% of CRCs have *APC* inactivation, which has a major role in adenoma formation, and subsequent multistage mutations such as *KRAS* and *TP53* mutations that cause carcinogenesis [8]. MSI is a hypermutable phenotype caused by abnormalities in DNA repair. Mismatch repair (MMR) proteins such as MLH1, MSH2, MSH6, and PMS2 are inactivated, and gene mutations accumulate. Lynch syndrome patients suffer from germline mutations in MMR-related genes, which induce tumors such as CRC [9]. Methylation in the promoter regions of MMR genes promotes suppression of MMR protein expression, defining a carcinogenesis pathway of CRC that differs from the classic adenoma–carcinoma sequence [10]. The overwhelming majority of these cases are caused by hypermethylation of the *MLH1* promoter [11].

Poorly differentiated (PD)-CRC has a poor prognosis compared with well and moderately differentiated CRC [12]. However, PD-CRC with MSI has a low lymph node metastasis rate and shows a good prognosis [13], although the mechanisms that define its clinicopathological differences have not yet been clarified. In this study, a new analysis focusing on *LGR5*-positive carcinoma stem cells was performed to investigate differences in prognosis based on a carcinogenesis model of PD-CRC. We also investigated the relationship between *LGR5* and β-catenin expression related to *LGR5* regulation, and that between CD8-positive tumor-infiltrating lymphocytes (CD8 + TILs) and *LGR5* expression in immune responses.

## Methods

### Patients and materials

A total of 625 CRC patients were selected at Shinshu University Hospital, Matsumoto, Japan from 2004 to 2014. PD-CRC was defined as the majority of the tumor being occupied by a PD-CRC component. All 29 PD-CRC cases were selected from the above patients. The clinicopathological features of these cases were evaluated.

### Histopathology, immunohistochemical staining, and evaluation

All samples were fixed in 8% formaldehyde and paraffin tumor blocks were made. Tumor blocks of CRC were selected to prepare a tissue microarray (TMA). The most representative region of each CRC sample was selected. Tissue cores were punched out from each block using thin-walled 3-mm stainless steel needles (Azumaya Medical Instruments Inc., Tokyo, Japan), and arrayed on a recipient paraffin block. Serial sections of 4-μm thickness cut from these blocks were stained with hematoxylin and eosin (HE) or immunostained with antibodies against MLH1 (ES05, mouse monoclonal; dilution, 1:50; Agilent Technologies, Santa Clara, CA, USA), PMS2 (EP51, rabbit monoclonal; dilution, 1:40; Agilent Technologies), MSH2 (FE11, mouse monoclonal; dilution, 1:50; Agilent Technologies), MSH6 (EP49, rabbit monoclonal; dilution, 1:50; Agilent Technologies), β-catenin (mouse monoclonal; dilution, 1:500; Becton-Dickinson & Company, Franklin Lakes, NJ, USA), or CD8 (CD8/144B, mouse monoclonal; dilution 1:50; Dako, Copenhagen, Denmark). For antigen retrieval, sections were boiled in 0.05% citraconic anhydride solution pH 7.4 (Immunosaver; Nissin EM, Tokyo, Japan) for 45 min for MLH1, PMS2, MSH2, and MSH6, or microwaved in 0.45% Tris/5 mM EDTA for 25 min for β-catenin and CD8. Detection of MMR proteins was performed using a NovoLink polymer detection system (Leica Microsystems GmbH, Wetzlar, Germany) and that of β-catenin and CD8 was performed using an Envision detection system (Agilent Technologies) according to the manufacturers’ recommendations.

In accordance with a previous report [14], the immunohistochemical staining for MLH1, PMS2, MSH2, and MSH6 was scored as positive when a nuclear staining pattern was observed. In addition, at least 5% of tumor cells in individual tissue cores were required to be stained. Cases of PD-CRC were determined to have MMR protein deficiency when at least one of MLH1, PMS2, MSH2, and MSH6 was negative.

β-Catenin staining was evaluated as previously described [15]. The results were calculated as IHC scores, where IHC score = percentage of nuclear positive cells × staining intensity. Nuclear staining was classified into five grades from 0 to 4. We defined staining intensity as follows: 0, negative; 1, weak; 2, moderate; 3, strong; and 4, very strong. The nuclear β-catenin IHC score ranged from 0 to 400. The number of CD8+ TILs was calculated in the three most infiltrated fields for each case using an intermediate-power field.

### *LGR5* RNA **in situ hybridization**

Detection of *LGR5* mRNA was performed with an RNAscope® kit (Advanced Cell Diagnostics, Hayward, CA, USA) according to the manufacturer’s instructions using unstained sample tissue slides. Briefly, tissue sections were pretreated by heating and protease was applied prior to hybridization with an *LGR5*-specific probe. The detailed procedure was described in a previous publication [16]. Brown dots present in the nucleus and/or cytoplasm were recognized as positive staining. *LGR5* expression was quantified using a five-level scoring system recommended by the manufacturer (0, no staining; 1, 1–3 dots/cell; 2, 4–10 dots/cell; 3, > 10 dots/cell; 4, > 15 dots/cell with > 10% of dots in clusters). The H-score was calculated as: (% of grade 1 cells × 1) + (% of grade 2 cells × 2) + (% of grade 3 cells × 3) + (% of grade 4 cells × 4). The overall H-score for each patient was calculated based on the H-score per high-power field (400× magnification). Furthermore, any cell with one or more dots was regarded as *LGR5*-positive.

### Statistical analysis

Statistical analysis was performed using JMP version 10 (SAS Institute Japan, Tokyo, Japan). Spearman’s rank correlation coefficient analysis was used to assess correlations. The Wilcoxon rank sum test or chi-square test was also applied to assess statistical significance. A value of *P* < 0.05 was considered significant.

## Results

### Correlations between clinicopathological factors and MMR protein expression

**Fig. 1:**
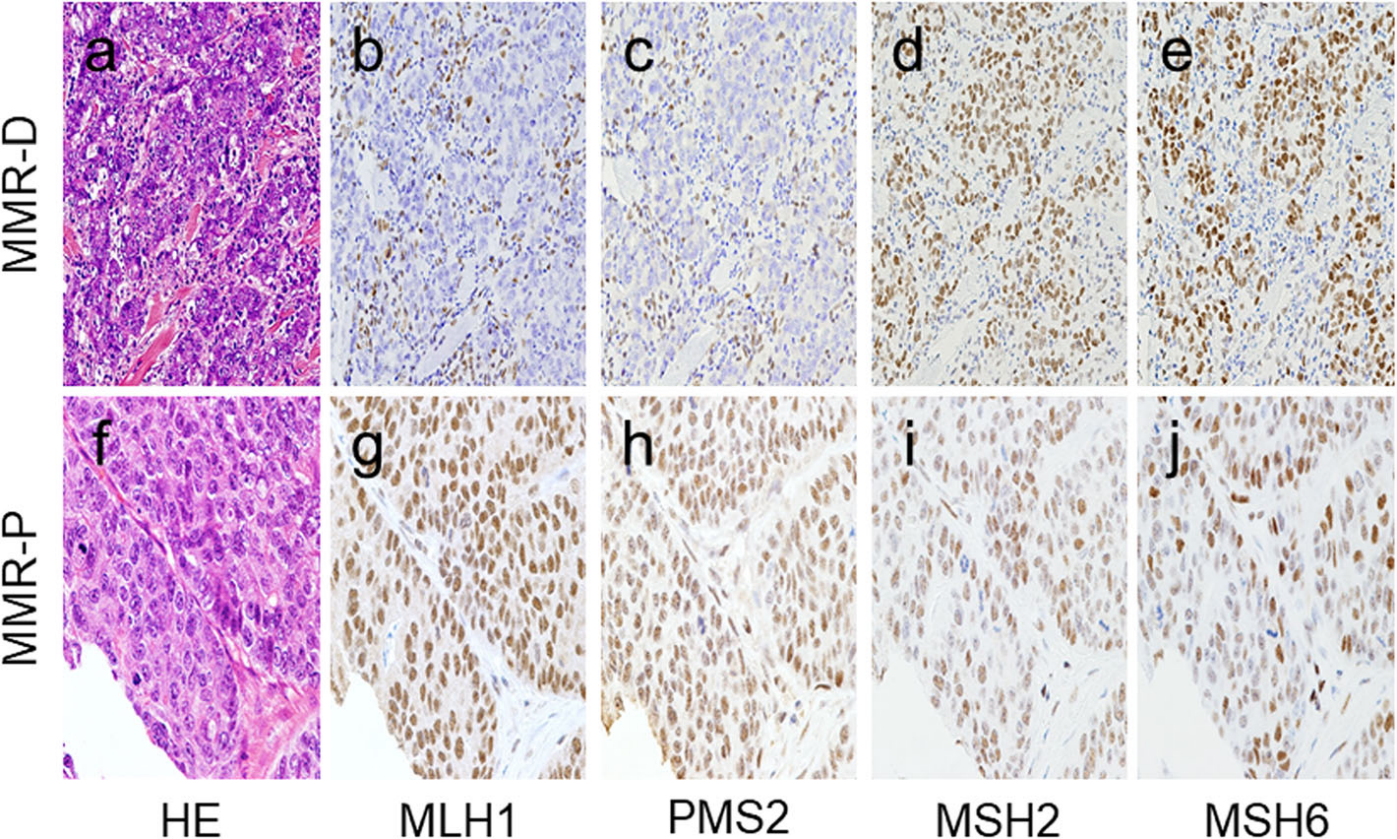
Representative images and staining of MMR-P and MMR-D cases. Representative features in MMR-D (**a**) and MMR-P (**f**). Immunohistochemistry of four MMR proteins. Loss of MLH1/PMS2 (**b** and **c**) and expression of MSH2/MSH6 (**d** and **e**) are shown in MMR-D. All four MMR proteins (**g**, **h**, **i**, and **j**) are detected in MMR-P. (**a** and **f**, HE; **b** and **g**, MLH1 immunostaining; **c** and **h**, PMS2 immunostaining; **d** and **i**, MSH2 immunostaining; **e** and **j**, MSH6 immunostaining)

**Fig. 2:**
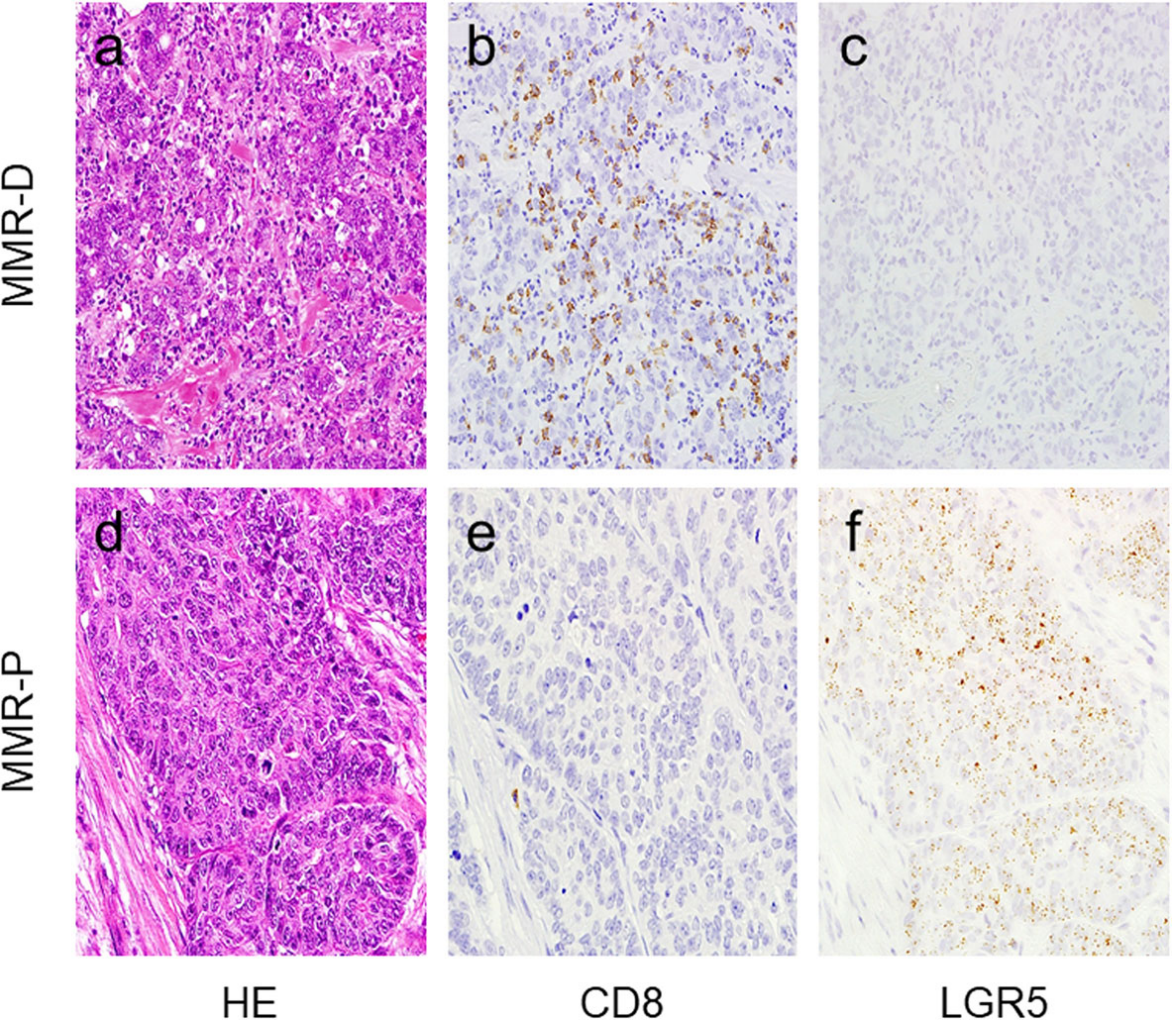
Representative images of *LGR5* and CD8 in MMR-P and MMR-D cases. Representative features in MMR-D (**a**) and MMR-P (**d**). In MMR-D, many CD8-positive lymphocytes were observed (**b**) and *LGR5* expression was low (**c**). In MMR-P, few CD8-positive lymphocytes were observed (**e**) and *LGR5* expression was high (**f**). (**a** and **d**, HE; **b** and **e**, CD8 immunostaining; **c** and **f**, *LGR5* RNAscope)

We first evaluated the expression of MMR proteins (MLH1, PMS2, MSH2, and MSH6) in 29 PD-CRC cases. Among the cases, 17 were MMR protein-proficient (MMR-P) and 12 were MMR protein-deficient (MMR-D) adenocarcinoma. In detail, 11 MMR-D cases showed dual loss of MLH1 and PMS2 and one MMR-D case showed dual loss of MSH2 and MSH6. Representative images and staining of MMR-D and MMR-P cases are shown in Fig. 1. Table 1 summarizes the correlations between clinicopathological factors and MMR protein expression. MMR-D cases were significantly correlated with age, tumor site, lymph node metastasis, pathological stage, and CD8+ TILs (Fig. 2a, b, d, e).

**Table 1:** Correlation between clinicopathological factors and MMR protein expression

	n	MMR protein		*P*-value
		Proficient	Deficient	
**Sex**				
Male	14	7	7	0.363
Female	15	10	5	
**Age**				
≥ 71	15	12	3	0.016*
< 71	14	5	9	
**Tumor site**				
Proximal	17	7	10	0.023*
Distal	12	10	2	
**Lymphovascular invasion**				
Absent	0	0	0	NA
Present	29	17	12	
**Lymph node metastasis**				
Absent	9	0	9	< 0.001*
Present	20	17	3	
**Pathological stage**				
≤ pT2	7	0	7	< 0.001*
> pT3	22	17	5	
**CD8+ TILs**				
≤ 155.7	15	15	0	< 0.001*
> 155.7	14	2	10	

*TIL* tumor-infiltrating lymphocytes

* *P* < 0.05

### *LGR5* RNA expression in PD-CRC with MMR protein deficiency

**Fig. 3:**
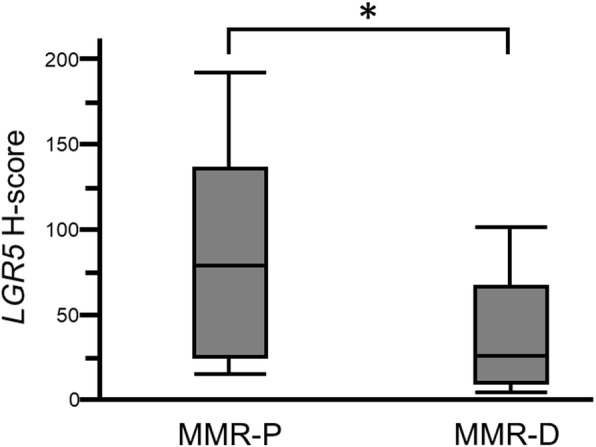
Box plot of *LGR5* H-scores in MMR-P and MMR-D. *LGR5* H-scores in MMR-D cases were significantly lower than those in MMR-P cases (*P* = 0.034)

We evaluated *LGR5* expression in all PD-CRC cases. All 29 cases contained carcinoma cells with some *LGR5*-positive dots, with a wide range of *LGR5*-positive cell staining. Representative images of *LGR5* staining in MMR-D and MMR-P cases are shown in Fig. 2c and f. *LGR5* H-scores varied among the cases. Mean H-scores for *LGR5* staining in MMR-P and MMR-D cases were 62.9 (24.2–136.2) and 24.4 (7.9–63.4), respectively. *LGR5* H-scores in MMR-D cases were significantly lower than those in MMR-P cases (*P* = 0.034) (Fig. 3).

### Correlation between *LGR5* expression and β-catenin expression

**Fig. 4:**
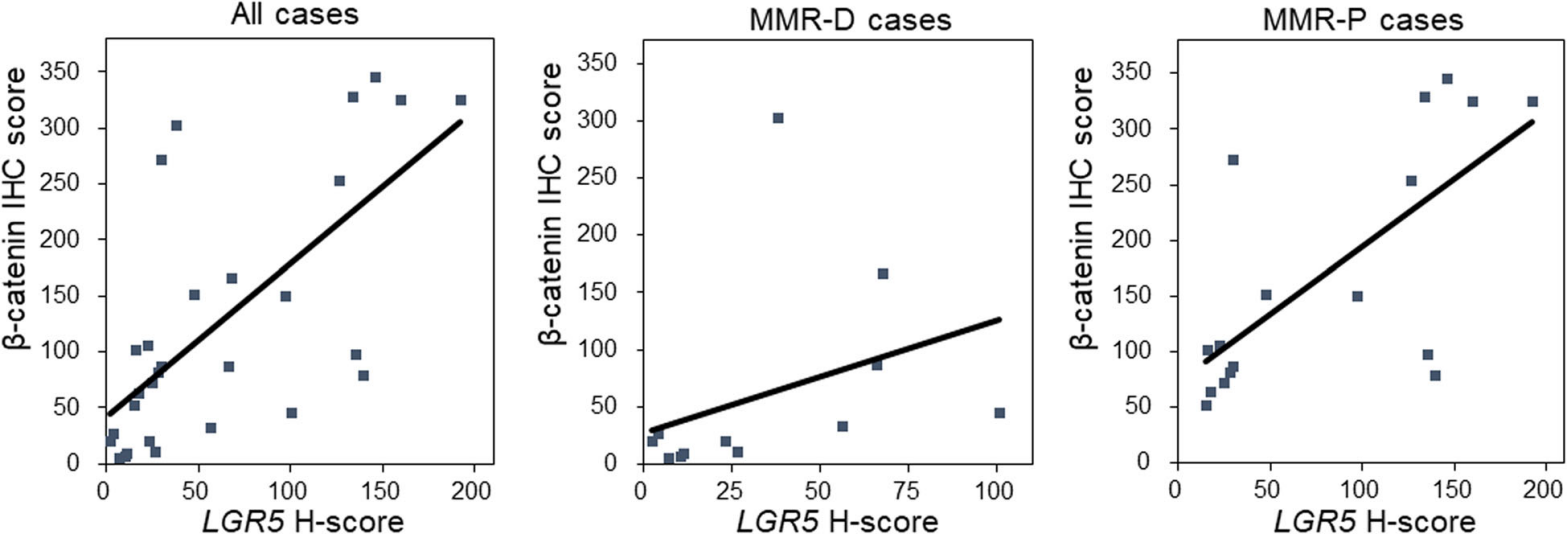
Scatter plots of *LGR5* H-score and nuclear β-catenin IHC scores. In all cases, MMR-D cases, and MMR-P cases, the scatter plots showed a positive correlation between *LGR5* H-score and nuclear β-catenin IHC score (r = 0.728, *P* < 0.001, r = 0.692, *P* = 0.013, and r = 0.679, *P* = 0.003, respectively)

Previous studies on CRC showed that β-catenin is related to *LGR5*, a cancer stem cell marker [17], and that β-catenin induces expression of *LGR5* [18]. We thus analyzed the correlation between *LGR5* H-score and expression of nuclear-translocated β-catenin. Mean nuclear β-catenin IHC scores in MMR-P cases and MMR-D cases were 104.5 (81.3–285.8) and 23.9 (9.9–77.1), respectively. Nuclear β-catenin IHC scores in MMR-D cases were significantly lower than those in MMR-P cases (*P* = 0.002). In all cases, there was a positive correlation between *LGR5* H-score and nuclear β-catenin IHC score (r = 0.728, *P* < 0.001) (Fig. 4). Even in MMR-D and MMR-P cases, there was a positive correlation between *LGR5* H-score and nuclear β-catenin IHC score (r = 0.692, *P* = 0.013 and r = 0.679, *P* = 0.003, respectively) (Fig. 4).

## Discussion

Most CRC cases show multistep carcinogenesis based on an adenoma–carcinoma sequence [19]. In the first step, inactivation of APC induces aberrant Wnt/β-catenin activation and contributes to tumorigenesis [19]. *LGR5* is the target gene of β-catenin and TCF/LEF complex, and nuclear-translocated β-catenin affects *LGR5* expression, as shown by previous reports and our current data [18, 20]. Deficiencies in MMR proteins are caused by genetic or epigenetic alterations in *MLH1*, *PMS2*, *MSH2*, and *MSH6*. Deficiencies in MMR proteins induce MSI and lead to the acquisition of various gene mutations [21]. In MSI carcinoma, genetic mutations such as *BRAF* mutations have been reported, while driver mutations frequently observed in CRC are infrequent [22]. In this study, morphologically similar CRC cases were classified into two biologically different groups based on the expression of MMR proteins, and their *LGR5* expression and clinicopathological features were analyzed.

Although the expression levels of *LGR5* differed between MMR-D and MMR-P cases, the correlation with β-catenin in both types of cases proves the association between β-catenin and *LGR5* expression. Low expression of *LGR5* in MMR-D cases may be related to low β-catenin expression. Furthermore, these cases may be linked to abnormalities related to Wnt/β-catenin signaling such as APC abnormalities.

There is controversy regarding the relationship between *LGR5* expression and prognosis in CRC. One issue is that MMR-D and MMR-P cases were pooled and analyzed together in previous studies. Another issue is the *LGR5* evaluation method. High expression of *LGR5* has been reported to be associated with poor prognosis, but this remains controversial. It may be because many prognostic evaluations are performed by immunohistostaining. In a report using the RNA in situ hybridization method, high expression of *LGR5* was observed in well differentiated CRC and showed better prognosis [4]. PD-CRC can arise through the adenoma–carcinoma sequence or the serrated neoplasia pathway. It has been pointed out that there is a difference in *LGR5* expression between the adenoma–carcinoma sequence and the serrated neoplasia pathway in RNA in situ hybridization [23] [24]. Because *LGR5* is a target of Wnt/β-catenin signaling that occurs during the adenoma–carcinoma sequence in CRC, *LGR5* expression in MMR-P cases, which may involve the adenoma–carcinoma sequence, may be higher than that in MMR-D cases, which may involve the serrated neoplasia pathway [25].

A recent report described differences in the presence of *LGR5*-positive carcinoma stem cells based on differences in MMR protein expression [4]. However, no studies have previously analyzed *LGR5* expression in detail in PD-CRC cases alone. In this study, we demonstrated that *LGR5* expression was significantly lower in MMR-D cases compared with MMR-P cases. The deficiency in MMR proteins contributed to an increase in tumor mutation burden in the tumor tissue. A characteristic of MSI carcinoma is the recruitment of infiltrating inflammatory cells due to increased antigen presentation by anti-cancer host immunity. A previous report [26] and our data also suggested that CD8+ TILs were present at high frequency in MMR-D CRC. The good prognosis of PD-CRC with MMR-D despite low expression of *LGR5* may be due to differences in immune responses. CRC should be divided into MMR-D cases involving the serrated neoplasia pathway and MMR-P cases involving the adenoma–carcinoma sequence if *LGR5* is used as a prognostic marker, and *LGR5* should be evaluated by RNA in situ hybridization. Analysis of a larger number of PD-CRC with MMR-D cases is warranted for more robust data. Although *LGR5* is a stem cell marker for CRC, classification into several subgroups may lead to *LGR5* being a more sensitive prognostic marker.

There are some limitations in our study. First, we did not examine mutations related to Wnt/β-catenin signaling such as APC, so these may need to be compared with *LGR5*. Furthermore, it is necessary to investigate the relationship between APC abnormalities and *LGR5* using cultured cells.

## Conclusions

Our results reveal a relationship between *LGR5* expression and MMR protein profiles in PD-CRC. A further study is warranted to confirm these findings.

## Data Availability

All data generated and analyzed during the current study are available from the corresponding author on reasonable request.

## Abbreviations

LGR5: Leucine-rich repeat-containing G-protein-coupled receptor 5
MMR: Mismatch repair
PD: Poorly differentiated
CRC: Colorectal carcinoma
IHC: Immunohistochemistry
MMR-D: MMR-deficient
MMR-P: MMR-proficient
MSI: Microsatellite instability
TILs: Tumor-infiltrating lymphocytes
TMA: Tissue microarray
